# Prediction of Mortality Incidence in Patients with Chronic Kidney Disease Based on Influential Prognostic Factors with Competing Risks Approach

**DOI:** 10.31661/gmj.v9i0.1798

**Published:** 2020-12-18

**Authors:** Anahita Saeedi, Ahmadreza Baghestani, Seyed-Saeed Hashemi-Nazari, Farzanehsadat Minoo, Navid Mohseni, Zahra Esfahani

**Affiliations:** ^1^Student Research Committee, Department of Biostatistics, Shahid Beheshti University of Medical Sciences, Tehran, Iran; ^2^Department of Biostatistics, Shahid Beheshti University of Medical Sciences, Tehran, Iran; ^3^Department of Epidemiology, School of Public Health, Shahid Beheshti University of Medical Science, Tehran, Iran; ^4^Center of Excellence in Nephrology, Nephrology Research Center, Tehran University of Medical Science, Tehran, Iran; ^5^Department of Biostatistics, Shahid Beheshti University of Medical Sciences, Tehran, Iran; ^6^Department of Biostatistics, University of Social Welfare and Rehabilitation Sciences, Tehran, Iran

**Keywords:** Chronic Kidney Disease, Survival Analyses, Competing Risks, Renal Transplantation

## Abstract

**Background:**

Chronic Kidney Disease (CKD) is a disease in which the kidney’s functionality declines gradually. The aim of this study was to identify significant laboratory prognostic factors on death due to CKD in a clinical complex.

**Materials and Methods:**

A retrospective study including 109 patients with the end-stage renal disease treated at Iran Helal pharmaceutical and the clinical complex was conducted between 2014-2018. The survival time was set as the time interval between starting dialysis until death due to CKD. Also, the transplantation was considered as competing risk, which was occurred for a few patients. A three-parameter Gompertz model was used that considers both the event of interest and the competing event simultaneously.

**Results:**

Death due to CKD occurred in 29 (26.6%) of the patients and 19(17.4%) with transplantation. Serum uric acid was a significant prognostic factor that decreased the hazard of mortality by 21%. Serum phosphorus and age by increasing the risk of death, were poor prognoses for the event of interest. Serum uric acid and phosphorus 6.9-9.9 (mg/dl) were associated with 72% and 4.05- fold increased hazard of transplant, respectively. The 4-year cumulative incidence of death and transplant was 48.4% and 29.2%, respectively.

**Conclusion:**

We have deduced that high serum phosphorus levels and increased levels of age were associated with worse outcomes. High serum uric acid level was related to better survival, which could be explained by having a better protein-rich diet alongside the high albumin level.

## Introduction

Chronic kidney disease (CKD) is a matter of healthcare associated with the slow loss of kidney function over time [1]. Generally, CKD is defined as either kidney damage (abnormalities observed during clinical assessment) or an estimated glomerular filtration rate (eGFR) less than 60 ml/min/1.73 m2 for at least three months [2]. In advanced stages of CKD, renal replacement therapy, including transplantation or dialysis, may be required [3]. The prevalence of CKD would perhaps increase gradually as a result of hypertension, glucose intolerance, obesity and hypercholesterolemia [4]. Knowledge about the prevalence and risk factors of this disease in different health jurisdictions could be useful in preventing early detection and optimized intervention and increasing survival time of patients with CKD [5]. Indeed, in most studies, the definition and specific information on the prognosis and risk factors responsible for CKD progression and death remains uncertain [6]. The incidence and prevalence of CKD are not known precisely since the disease does not appear to be symptomatic in its early stages; however, the prevalence was estimated around 10% to 14% in the world [7]. Also, it has been estimated that more than 500 million people have CKD, which 80% of them are living in the less developed countries [8]. Globally, the incidence of CKD increased significantly in the last three decades, and the incidence rate increased slightly from about 214 (per 100,000 population) in 1990 to about 288 (per 100,000 population) in 2016 [9]. In Iran, the last estimation of prevalence was 15.4% based on a systematic review that was higher for females than males [10]. Over the past decade, the incidence rate in Iran was estimated at about 208 (per 10,000), and it has been rising ever since [11]. Worldwide, the deaths from CKD in 1990 and 2016 were estimated at about 500,000 cases and 1,100,000, respectively, indicating a significant increase in mortality rate during these 27 years. Besides, the mortality rate of CKD per 100,000 population was estimated at about 16.05 in 2016, which is 1.4 times higher than the rate in 1990 [9]. In Iran, the CKD mortality rate was estimated at over 2% based on the global burden of disease, which is twice the mortality rate in the last decade [12,13]. Mainly, the survival of CKD patients with dialysis is affected by diseases such as cardiovascular and high blood pressure, and the 5-year survival rate for these patients is estimated at 34% that is not very high [14]. However, there is no estimation for the survival of CKD patients in Iran. Regular dialysis is a common treatment method for improving the survival of CKD patients. Another method is kidney transplant, which could be performed for CKD patients with eGFR less than 15 ml/min/1.73m2 [14]. In this stage, the mortality rate of CKD is higher than in the other stages, and the primary way to reduce the death rate is transplantation [15,16]. Therefore, the probability of death may be altered due to other events such as renal transplantation, known as a competing risk. In medical studies, the survival analysis is defined as the time until certain events occur (such as death due to CKD). However, some patients (CKD patients) could experience other events (such as transplantation), which prevents us from observing the event of interest. In such cases (also known as competing risk), the Kaplan-Meier method and Cox regression could not be appropriate since they produce biased estimations [17]. In the current study, we deployed a 3-parameter Gompertz distribution [18] for the survival of CKD patients with transplantation as a competing risk in order to estimate more precise survival probabilities

## Materials and Methods

###  Patients

 In this retrospective cohort study, a total of 109 hemodialysis patients were enrolled and registered in the Iran Helal pharmaceutical and clinical complex during 2014-2018. The inclusion criteria were age higher than 18 years old, and routine dialysis started at least three months before the study. The exclusion criteria were evidence of acute kidney injury and acute illness. Demographics and laboratory results were collected from hospital medical records and included all CKD patients given hemodialysis three times a week. The survival time was calculated as the time interval between starting dialysis and time of death due to CKD. The transplantation was considered as a competing risk. The study was confirmed by the ethical committee of Shahid Beheshti University of Medical Sciences, Tehran, Iran (approval code:IR.SBMU.RETECH.REC.1398.282).

###  Measurements 

 At the beginning, information on demographics was collected. A blood sample was drawn after 12–14 hours on an empty stomach and was centrifuged within 30–45 min of collection. The analyses of the blood tests were completed at the reference laboratory on the day of sampling. Total cholesterol was measured using the enzymatic colorimetric method with cholesterol esterase-cholesterol oxidase. Serum creatinine levels were evaluated by kinetic colorimetric Jaffe. Serum uric acid, serum glutamic oxaloacetic transaminase (SGOT), serum glutamic-pyruvic transaminase (SGPT), bilirubin, hemoglobin, potassium, Alkaline phosphatase (ALP), hemoglobin A1c (HbA1C), ferritin, calcium, serum phosphorus, parathyroid hormone (PTH) and albumin were also measured. All biochemical tests were carried out using commercial kits (Pars Azmoon Inc., Tehran, Iran) by a Selectra 2 auto-analyzer (Vital Scientific, Spankeren, the Netherlands).

###  Statistical Analysis

Descriptive statistics (mean standard deviation (SD) for continuous variables and frequency for categorical factors) were used to summarize demographic and prognostic variables in the study of the population. The quantitative variables were classified based on the criteria in the handbook of dialysis [19]. The categorizations were then accepted and finalized by the participating nephrologist. The 3-parameter Gompertz model [18] was applied, which considers the event of interest and the competing event simultaneously. The cumulative incidence and survival probabilities of events (including death and transplant) were estimated. The univariate and multivariate effect of variables on the survival time and transplant were evaluated as well. Our primary outcome measure was whether the independent prognostic variables have any effect on the mortality due to CKD. The results are presented as the sub-hazard ratio (S-HR) from the 3-parameter Gompertz model with competing risk. Moreover, 75% and 90% confidence interval (CI) were presented for the estimated effect in the models, respectively. All statistical analyses were conducted using SAS version 9.4 and the R programming language version 3.5.2. The P-value<0.25 and P-value<0.1 were considered statistically significant for univariate and multivariate models, separately.

## Results

**Figure 1:**
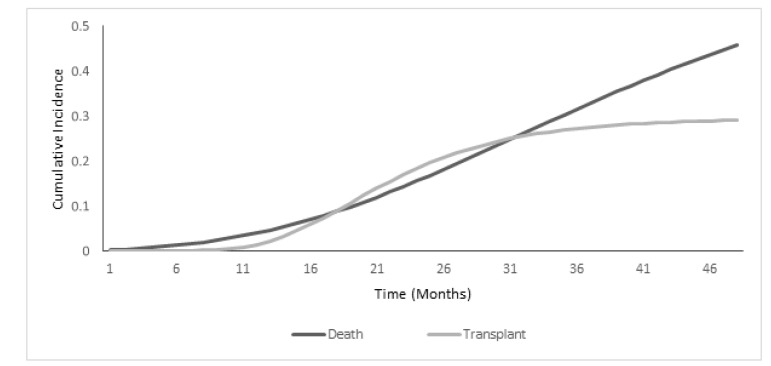
The Cumulative incidence curve of death due to CKD and transplant. The 1, 2, 3 and 4-year incidence of both events are presented.

 The analysis was performed on 109 hemodialysis patients (71.6% male and 28.4% female) with mean age of 57.99 17.10. Overall, 29 (26.6%) of the patients experienced death due to CKD and others were censored (Table-1). The one, two, three, and four-year cumulative incidence for CKD patients with death (transplant) were 2.5% (1.34%), 14.2% (22.6%), 32.6% (28.6%) and 48.4% (29.2%), respectively (Table-2). At the end of the study, as it is shown in Figure-1, the cumulative incidence of death is about 50%, which is higher than 30% of the cumulative incidence of transplant. The Kt/V (an index of the removal efficiency per dialysis session) had no significant difference among all the patients, and it was 1.2± 0.2. In the next step, for estimation of the S-HR, the 3-parameter Gompertz model was applied. Results indicated that in the univariate analysis, older age, being male, high cholesterol, serum uric acid, SGPT and bilirubin, hemoglobin >12.5 g/dl, ALP >300 U/L, serum phosphorus between 4.7-5.7 and 6.9-9.9 mg/dl, PTH <150 pg/dl, and albumin 4-6.3 g/dl had a significant effect on the survival of CKD patients when the event of interest (death) was considered as an outcome (P<0.25). In the multivariate analysis, assuming that the effects of all the other variables were constant, the adjusted S-HR for age was 1.02 (90% CI=1.01-1.03, P=0.000), which indicates that for a one-year increase in age, the hazard of death increases by 2%. Furthermore, for a one-milligram increase in serum uric acid, the risk of death for hemodialysis patients was decreased by 21% (90% CI=0.68-0.91, P=0.007). The adjusted effect of serum phosphorus was significant on the survival of hemodialysis patients, and for those with serum phosphorus between 4.7-5.7 mg/L, the hazard of death was 49% higher compared to patients with serum phosphorus between 2.7-4.6 mg/L (90% CI=1.02-2.17, P=0.082). When the transplant was the outcome, the univariate analysis demonstrated that high ferritin, cholesterol, serum uric acid, calcium >9.5mg/dl, serum phosphorus 5.8-6.8 and 6.9-9.9 mg/dl, PTH<150 pg/dl, and albumin 4-6.3 g/dl had a significant effect on the transplant of hemodialysis patients (P<0.25). In the multivariate analysis, age, serum uric acid, gender and serum phosphorus were presented as influential risk factors. Holding the effect of all the other variables constant, the hazard of receiving a kidney transplant decreased by 4% for a one-year increase in age (90% CI=0.95-0.97, P<0.001). The hazard of receiving a kidney transplant was 46% higher for males than females (90% CI=1.002-2.14, P=0.097). Moreover, for a one-milligram increase in serum uric acid, the hazard of receiving a transplant increased by 72% (90% CI=1.47-2.00, P<0.001). For patients with serum phosphorus levels between 6.9-9.9 mg/dl, the hazard of having a transplant was 4-fold higher compared to patients with the lowest level of serum phosphorus (90% CI=2.46-6.66, P<0.001, Table-3).

## Discussion

Designating the right statistical approach for determining the effect of prognostic factors is very significant [20]. In this analysis, using the 3-parameter Gompertz model, we observed that the variables such as age, serum uric acid, gender and serum phosphorus between 4.7-5.7 and 6.9-9.9 mg/L had a significant adjusted effect on the time until death\transplant occurs. In our dataset, more than half of the patients survived until the end of the study, and about 70% did not have kidney transplants. The study outcomes reveal that males have a higher hazard of transplant compared to females. No significant association was found between being male and a higher risk of mortality. However, the male gender was most often related to a higher risk of CKD progression, especially in the end-stage of the disease [21]. Carrero *et al*. illustrated that despite the higher prevalence of CKD in females, hemodialysis patients’ mortality rate was higher for males [17]. The hazard of the transplant was also higher in men compared to women [22]. Other studies found that the risk of kidney transplantation has been higher in males than females [23-25]. These differences may be due to the distinct biological and behavioral characteristics of males and females.

Studies showed that the prevalence of CKD in all stages was higher in elderly patients [26]. However, older patients were less likely to have kidney transplants [27,28], and access to kidney transplantation decreased with old age [29], which was consistent with our result. Contrarily, the risk of death increased in older ages. These findings are in concordance with the results of former studies [15,30,31]. Kidney function and the survival rate of patients after transplantation are some of the most important medical issues. A study conducted by Meier-Kriesche, *et al*. illustrated that low renal function was linked to higher serum uric acid levels, which leads to a lower survival rate [32]. In other studies by Chonchol *et al*. and Rincon *et al*., serum uric acid was strongly associated with increased risk of progression to end-stage renal disease in which the survival rate was lower than early stages [33,34]. In our study, increasing serum uric acid was connected with a higher risk of having a kidney transplant and a lower risk of death. In this dataset, higher serum uric acid correlated with higher albumin. Since malnutrition is greatly conjoined with mortality, we can conclude that patients with a protein-rich diet have healthier and better nutrition, which could be linked to higher serum uric acid levels and a decrease in the risk of mortality. The results of the multivariable model for transplant revealed that patients with higher serum uric acid are more at the risk of transplantation. In studies by Go *et al*, and Hruska *et al*., hyperphosphatemia had led to CKD, which was associated with excess mortality [35,36]. Our study demonstrated that patients with serum phosphorus level 6.9-9.9 (mg/L) had the risk of having a transplant 4-fold greater than patients with the lowest level of serum phosphorus. Also, higher serum phosphorus level (4.7-5.7) was associated with a higher risk of mortality compared to lower levels (2-7-4.6). This implies that even in earlier stages of renal dysfunction, low serum phosphorus level is related to poor prognosis. However, intervention trials are required to study whether lowering phosphate will improve patients’ survival with earlier CKD stages.

###  Limitations

 There were some limitations in our study, such as the small sample size, which could have reduced the power of the test. The other issue was multicollinearity that made it impossible to use all significant variables in the multivariate model.

## Conclusion

 We have found that patients with relatively high phosphate levels were associated with a worse outcome than those with serum phosphate in lower ranges. Our study pointed out that higher serum uric acid level was connected with a lower risk of mortality. Moreover, we have demonstrated that the 3-parameter Gompertz model was more flexible for the evaluation of the significant laboratory prognostic factors compared to cause-specific and subdistribution models since it considers the event of interest and the competing event simultaneously. However, further investigations are required with larger sample sizes for reaching accurate conclusions.

## Acknowledgment

 This study is related to project No. 1398.10495 from the Student Research Committee, Shahid Beheshti University of Medical Sciences, Tehran, Iran. We also appreciate the Student Research Committee and Research & Technology Chancellor at Shahid Beheshti University of Medical Sciences for their financial support of this study. The information needed for this research was provided with the help of the Iran Helal pharmaceutical and clinical complex.

## Conflict of Interest

 The authors declare that there is no conflict of interest regarding the publication of this article.

**Table 1:** Demographic and Prognostic Factors of the Study Population.

**Characteristics**	**(%) Frequency/ Mean SD**		
	**total**	**death**	**transplant**
**Age(years)**	57.99±17.10	72.00±8.10	49.47±17.99
**Ferritin(ng/ml)**	347.90±325.43	436.48±334.33	278.76±307.40
**Creatinine(mg/dl)**	8.93±3.61	7.66±3.22	8.10±4.27
**Cholesterol(mg/dl)**	146.64±36.47	137.78±27.41	153.20±45.65
**Uric Acid(mg/dl)**	7.00±1.33	6.70±0.90	7.07±1.27
**SGOT(U/L)**	18.40±7.53	19.82±8.37	18.44±5.01
**SGPT(U/L)**	21.33±13.97	20.95±8.16	22.36±11.56
**Bilirubin(mg/dl)**	0.68±0.10	0.68±0.09	0.71±0.19
**Hemoglobin(g/dl)**			
<10	37(33.9)	9(31.0)	7(36.8)
10-12.5	47(43.1)	11(37.9)	8(42.10)
>12.5	25(22.9)	9(31.0)	4(21.1)
**Gender**			
Male	78(71.6)	27(93.1)	16(84.2)
Female	31(28.4)	2(6.9)	3(15.8)
**Potassium( mEq /L)**			
>5	73(67.0)	17(58.6)	11(57.9)
3.5-5	36(33.0)	12(41.4)	8(42.1)
**ALP(U/L)**			
>300	50(45.9)	13(44.8)	10(52.6)
<300	59(54.1)	16(55.2)	9(47.4)
**HBA1c(mmol/mol)**			
>7	13(11.9)	3(10.3)	3(15.8)
<7	96 (88.1)	26(89.7)	16(84.2)
**Calcium(mg/dl)**			
<8.5	15(13.8)	6(20.7)	0(0.0)
8.5-9.5	80(73.4)	17(58.62)	17(89.5)
>9.5	14(12.8)	6(20.7)	2(10.5)
**Phosphorus(mg/dl)**			
2.7-4.6	5(4.6)	10(34.5)	5(26.3)
4.7-5.7	21(19.3)	7(24.1)	10(52.6)
5.8-6.8	56 (51.4)	8(27.6)	3(15.8)
6.9-9.9	27(24.8)	4(13.8)	1(5.3)
**PTH( pg /dl)**			
150-600	84(77.1)	22(75.9)	14(73.7)
<150	14(12.8)	6(20.7)	4(21.1)
>600	11(10.1)	1(3.4)	1(5.3)
**Albumin(g/dl)**			
4-6.3	85(78)	21(72.4)	16(84.2)
<4	24(22)	8(27.6)	3(15.8)

**SD: **standard deviation; **SGOT: **serum glutamic-oxaloacetic transaminase; **SGPT:** serum glutamic-pyruvic transaminase; **ALP:** Alkaline Phosphatase; **HBA1c: **Hemoglobin A1c; **PTH:** parathyroid hormone

**Table 2:** Cumulative Incidence Probabilities of Events

**Year cumulative incidence**	**Death( %)**	**Transplant (%) **
**1-year **	2.5	1.34
**2-year **	14.2	22.6
**3-year **	32.6	28.6
**4-year **	48.4	29.2

**Table 3:** Univariate and Multivariate Competing Risks Model of 109 Cases of Chronic Kidney Disease

**Variables**		**Univariate**		**Multivariate**	
	**Subgroup**	**S-HR (75% CI)**	**P-value**	**Adjusted S-HR (90% CI)**	**P-value**
**Age **(years) _(Death)_	-	1.01(1.007-1.07)	0.047*	1.02(1.01-1.03)	<0.001*
**Ferritin **(ng/ml) _(Death)_	-	0.99(0.99-1.00)	0.747		
**Creatinine **(mg/dl)_ (Death)_	-	1.03(0.94-1.14)	0.492		
**Cholesterol **(mg/dl) _(Death)_	-	0.98(0.98-0.99)	0.009*		
**Uric acid **(mg/dl)_ (Death)_	-	0.78(0.64-0.95)	0.014*	0.79(0.68-0.91)	0.007*
**SGOT **(U/L) _(Death)_	-	1.01(0.97-1.05)	0.387		
**SGPT **(U/L) _(Death)_	-	1.02(1.006-1.04)	0.127*		
**‌ Bilirubin **(mg/dl)_ (Death)_	-	17.46(1.09-2.55)	0.087*		
**Gender **_(Death)_	Male	2.03(1.38-2.97)	0.031*	1.59(0.97-2.62)	0.120
	Female	-	1		
**Hemoglobin **(g/dl) _(Death)_	<10	0.78(0.52-1.16)	0.485		
	10-12.5	-	1		
	>12.5	0.52(0.36-0.76)	0.050*		
**Potassium **(mEq/L) _(Death)_	>5	0.90(0.54-1.51)	0.829		
	3.5-5	-	1		
**ALP **(U/L) _(Death)_	>300	1.60(1.08-2.38)	0.164*		
	<300	-	1		
**HbA1C **(mmol/mol) _(Death)_	>7	0.59(0.32-1.09)	0.324		
	<7	-	1		
**Calcium** (mg/dl)_ (Death)_	<8.5	1.50(0.96-2.33)	0.288		
	8.5-9.5	-	1		
	>9.5	0.80(0.51-1.24)	0.566		
**Phosphorus **(mg/dl) _(Death)_	2.7-4.6	-	1		
	4.7-5.7	2.20(1.32-3.66)	0.076*	1.49(1.02-2.17)	0.082**
	5.8-6.8	0.65(0.40-1.03)	0.290	1.06(0.66-1.69)	0.826
	6.9-9.9	0.46 (0.31-0.69)	0.290*	0.60(0.33-1.08)	0.154
**PTH **(pg/dl) _(Death)_	150-600	-	1		
	<150	1.61(1.07-2.43)	0.178*		
	>600	0.65(0.28-1.50)	0.559		
**Albumin **(g/dl) _(Death)_	4-6.3	0.61(0.41-0.90)	0.150*		
	<4	-	1		
**Age **(years) _(Transplant)_	-	0.99(0.99-1.01)	0.813	0.96(0.95-0.97)	<0.001*
**Ferritin **(ng/ml) _(Transplant)_	-	0.91 (0.08-0.99)	0.027*		
**Creatinine **(mg/dl) _(Transplant)_	-	0.99 (0.99-1.00)	0.747		
**Cholesterol **(mg/dl) _(Transplant)_	-	1.00 (1.001-1.01)	0.016*		
**Uric** **Acid** (mg/dl) _(Transplant)_	-	1.36 (1.13-1.63)	0.001*	1.72 (1.47-2.00)	<0.001*
**SGOT **(U/L) _(Transplant)_	-	0.99 (0.95-1.03)	0.923		
**SGPT **(U/L) _(Transplant)_	-	0.99 (0.98-1.01)	0.650		
**‌ Bilirubin **(mg/dl) _(Transplant)_	-	0.10 (0.01-1.10)	0.270		
**Gender **_(Transplant)_	Male	0.87 (0.54-1.40)	0.747	1.46(1.002-2.14)	0.097*
	Female	1	-	1	-
**Hemoglobin **(g/dl) _(Transplant)_	<10	0.93 (0.62-1.38)	0.837		
	10-12.5	-	1		
	>12.5	1.38(0.84-2.27)	0.453		
**Potassium **(mEq/L) _(Transplant)_	>5	1.25 (0.75-2.08)	0.613		
	3.5-5	-	1		
**ALP **(U/L) _(Transplant)_	>300	0.70 (0.43-1.12)	0.473		
	<300	-	1		
**HbA1C **(mmol/mol) _(Transplant)_	>7	1.27 (0.38-4.26)	0.811		
	<7	-	1		
**Calcium **(mg/dl) _(Transplant)_	>9.5, <8.5	0.46(0.21-1.01)	0.255		
	8.5-9.5	-	1		
	>9.5	1.91(1.03-3.55)	0.225*		
**Phosphorus **(mg/dl) _(Transplant)_	2.7-4.6	-	1		
	4.7-5.7	0.67 (0.34-1.27)	0.474	0.85(0.62-1.17)	0.416
	5.8-6.8	2.77 (1.44-5.31)	0.071*	1.23(0.88-1.74)	0.298
	6.9-9.9	8.08 (3.97-16.44)	<0.001*	4.05(2.46-6.66)	<0.001*
**PTH **(pg/dl) _(Transplant)_	150-600	-	1		
	<150	0.50 (0.32-0.77)	0.069*		
	>600	0.99 (0.37-2.63)	0.996		
**Albumin **(g/dl) _(Transplant)_	4-6.3	1.52 (1.09-2.91)	0.168*		
	<4	-	1		

* Significant at 0.25, ** Significant at 0.10 **S-HR:** Standard hazard ratio; **CI:** Confidence Interval; **SGOT: **Serum glutamic-oxaloacetic transaminase; **SGPT:** Serum glutamic-pyruvic transaminase; **ALP:** Alkaline phosphatase; **HbA1C:** Hemoglobin A1c; **PTH:** Parathyroid hormone
